# Placental chemokine compartmentalisation: A novel mammalian molecular control mechanism

**DOI:** 10.1371/journal.pbio.3000287

**Published:** 2019-05-29

**Authors:** Kit Ming Lee, Gillian J. Wilson, Marieke Pingen, Ayumi Fukuoka, Christopher A. H. Hansell, Robin Bartolini, Laura Medina-Ruiz, Gerard J. Graham

**Affiliations:** Chemokine Research Group, Institute of Infection, Immunity and Inflammation, University of Glasgow, Glasgow, United Kingdom; Yale University, UNITED STATES

## Abstract

Atypical chemokine receptor 2 (ACKR2) is a chemokine-scavenging receptor. ACKR2–/–embryos display a reduction in size of a novel, to our knowledge, embryonic skin macrophage population referred to as ‘intermediate’ cells. CC chemokine receptor 2 (CCR2)–/–embryos display an identical phenotype, indicating that these cells require CCR2 to enable them to populate embryonic skin. Further analysis revealed that ACKR2–/–embryos have higher circulating concentrations of the CCR2 ligand, CC ligand 2 (CCL2); thus, ACKR2 regulates intraembryonic CCL2 levels. We show that ACKR2 is strongly expressed by trophoblasts and that it blocks movement of inflammatory chemokines, such as CCL2, from the maternal decidua into the embryonic circulation. We propose that trophoblastic ACKR2 is responsible for ensuring chemokine compartmentalisation on the maternal decidua, without which chemokines enter the embryonic circulation, disrupting gradients essential for directed intraembryonic cell migration. Overall, therefore, we describe a novel, to our knowledge, molecular mechanism whereby maternal decidual chemokines can function in a compartmentalised fashion without interfering with intraembryonic leukocyte migration. These data suggest similar functions for other atypical chemokine receptors in the placenta and indicate that defects in such receptors may have unanticipated developmental consequences.

## Abstract 2

A novel mechanism for molecular compartmentalisation in the placenta involves an atypical chemokine receptor that scavenges chemokines, blocking their drainage from the maternal face of the placenta into the embryo and thus protecting intraembryonic cellular migration processes.

## Introduction

There is considerable literature on the regulation of migration of discrete leukocyte populations, which is predominantly controlled by members of the chemokine family [1]. This family of small proteins is defined on the basis of a conserved cysteine motif and is divided into CC, CXC, XC, and CX3C subfamilies according to the specific configuration of this motif. Chemokines interact with target cells through receptors belonging to the 7-transmembrane–spanning family of G-protein–coupled receptors [2]. These receptors direct migration of specific leukocyte subtypes and, for example, the key chemokine receptors regulating monocyte/macrophage migration are CC receptor 2 (CCR2) and CX3C receptor 1 (CX3CR1) [3]. Chemokines and their receptors therefore represent the major regulators of leukocyte migration and can be characterised as being either inflammatory or homeostatic according to the contexts in which they function. In addition to the classical signalling chemokine receptors, there exists a small subfamily of atypical chemokine receptors [4–6]. These molecules are 7-transmembrane–spanning receptors, but they differ from the other chemokine receptors in that they do not mount classical signalling responses following ligand binding and are generally expressed on stromal populations, where they play roles in fine-tuning chemokine-driven responses. We have a particular interest in one of these atypical receptors, atypical chemokine receptor 2 (ACKR2), which promiscuously binds inflammatory CC-chemokines and targets them for intracellular degradation [5, 7]. It has thus been proposed to be a scavenging receptor for inflammatory CC-chemokines. We, and others, have demonstrated essential roles for ACKR2 in the regulation of inflammatory responses and the functional implications of this for the development of a variety of inflammatory pathologies and cancers [8–11]. In addition, we have demonstrated roles for ACKR2 and CCR2 in regulating macrophage-dependent developmental processes [12, 13]. Notably, ACKR2 is specific to mammals and is strongly expressed in the foetal-derived trophoblast layer of the placenta. To date, we have a limited understanding of its role in this biological context and of the reason for its mammalian specificity.

Here, we report a major and unanticipated developmental phenotype in which ACKR2–/–embryonic skin displays a selective reduction in the size of a novel, to our knowledge, macrophage population. CCR2–/–mice display an identical phenotype, indicating that this embryonic skin macrophage population positions itself within the embryo in a CCR2-dependent manner. This population is characterised by intermediate expression of CD11b and F4/80 and is transcriptionally distinct from previously characterised embryonic monocyte/macrophage subtypes [14]. We further demonstrate that the impaired development of this population in ACKR2–/–embryos is a result of lack of ACKR2-dependent placental chemokine compartmentalisation. This results in the passage of chemokines from the maternal decidua to the developing embryo and disruption of intraembryonic chemokine gradients essential for correct tissue-specific migration of this novel, to our knowledge, macrophage population. Our data therefore demonstrate a fundamental role for placental ACKR2 in protecting the intraembryonic migration of a CCR2-dependent subpopulation of macrophages during development and highlight placental chemokine compartmentalisation as an important and novel, to our knowledge, mechanism for enabling accurate intraembryonic leukocyte migration.

## Results

### ACKR2–/–skin displays deficiencies in a monocyte/macrophage population

**Fig 1:**
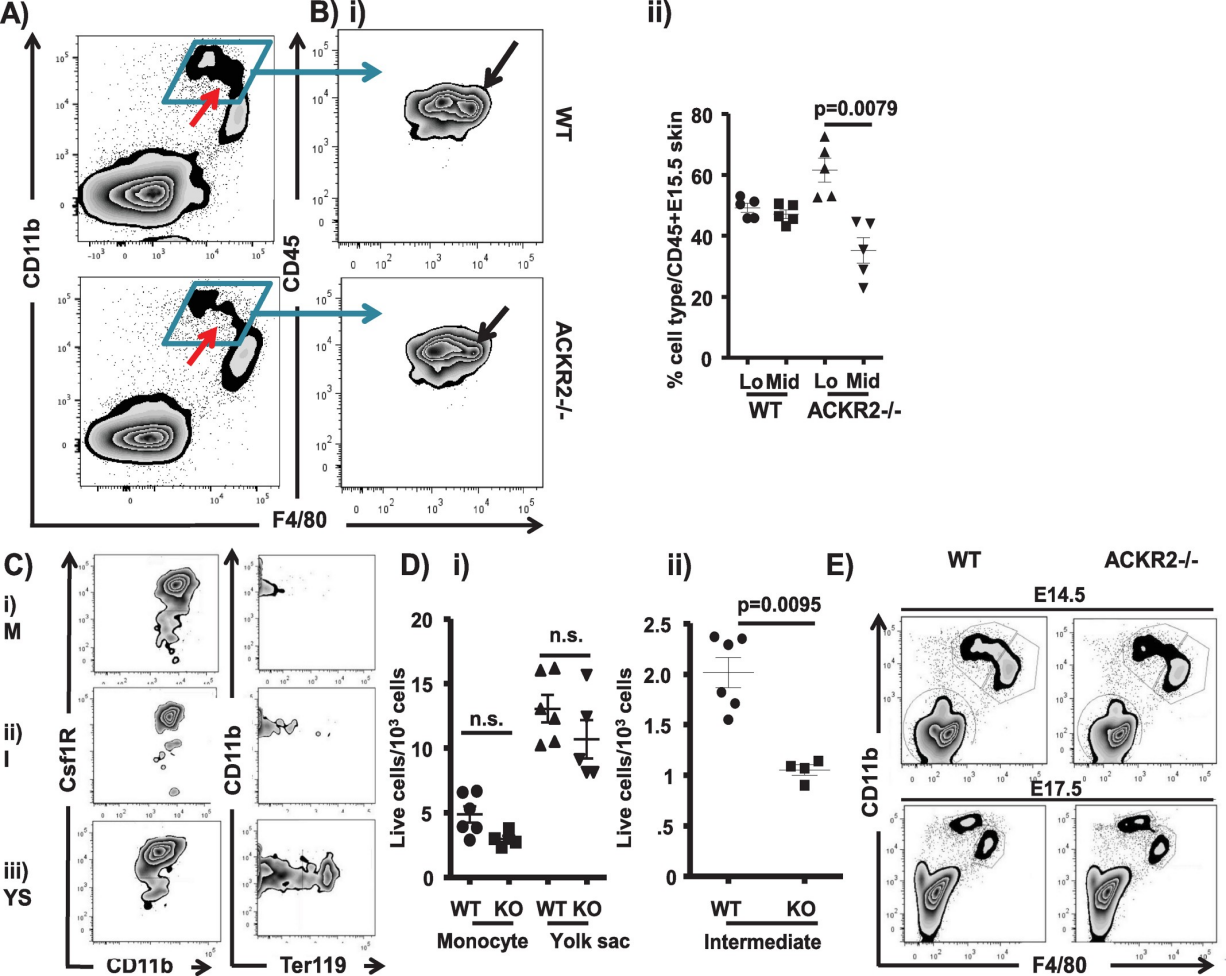
Monocyte/macrophage deficiency in ACKR2–/–embryonic skin. A) Flow cytometry plot showing CD11b/F4/80 staining of CD45+ myelomonocytic cells in E15.5 WT and ACKR2–/–skin. The red arrow marks the intermediate population. B) i) Flow cytometric analysis of CD45/F4/80 staining in the total CD11bhi gate from skin of E14.5 WT (left-hand plot) and ACKR2–/–embryos (right-hand plot), showing a selective reduction in the F4/80Mid population (arrowed). ii) Quantitation of the percentage of F4/80lo and F4/80mid cells in WT and ACKR2–/–(KO) E15.5 embryonic skin. Results are representative of 2 separate experiments. Mann–Whitney U test was used for statistical analysis. C) Flow cytometric assessment of CSF1R and Ter119 staining of the i) monocyte (M), ii) intermediate (I), and iii) yolk-sac (YS)–derived cellular populations from E14.5 skin of MacGreen embryos. D) Quantitative assessment of multiple flow cytometric analyses showing numbers of i) monocyte- and yolk-sac–derived and ii) intermediate cells in E14.5 WT and ACKR2–/–(KO) skin. Results are from 1 of 4 independent experiments. Statistical analysis used the Mann–Whitney U test. E) Flow cytometric analysis of CD11b/F4/80 expression by CD45+ myelomonocytic cells from WT and ACKR2–/–(KO) skin at E14.5 and E17.5. Data are representative of at least 2 repeat experiments. Data were pooled from 3 independent experiments. Mann–Whitney test (monocytes) and unpaired two-tailed Student *t* test (yolk-sac–derived macrophages) were used for statistical analysis. Data associated with this figure can be found in the supplemental data file (S2 Data). ACKR, atypical chemokine receptor; CSF1R, Colony-stimulating factor 1 receptor; E, embryonic day; KO, knockout; WT, wild type.

Embryonic monocyte/macrophage populations are classically characterised as being either monocyte (CD11bhiF4/80lo) or yolk sac (YS) (CD11bloF4/80hi) derived [14–18] (S1 Fig, panel A). As shown in Fig 1A and as previously reported [12], flow cytometric analysis of monocyte/macrophage populations in skin from embryonic day (E) 15.5 wild-type (WT) and ACKR2–/–embryos revealed a clear and significant reduction in the overall size of the CD11bhiF4/80lo ‘monocyte’ gate in ACKR2–/–mice. In contrast, no differences were detected in the population of CD11bloF4/80hi yolk-sac–derived macrophages. Within the overall CD11bhiF4/80lo gate, further analysis gating on CD45 and F4/80 revealed overlapping F4/80lo and F4/80mid populations of cells (Fig 1Bi). Notably, comparison of WT and ACKR2–/–embryonic skin revealed a specific and significant reduction in the subpopulation of CD11bhiF4/80mid cells in the ACKR2–/–embryos with retention of the population of CD11bhiF4/80lo cells (Fig 1Bi and 1Bii). This population of cells therefore sits intermediate between the monocyte and yolk-sac–derived populations (red arrow in Fig 1A), and they are henceforth referred to as ‘intermediate’ cells. These cells are characterised by strong expression of the Colony-stimulating factor 1 receptor (CSF1R) and lack of expression of Ter119 [19] (Fig 1C), and they display medium granularity and size as indicated by forward and side scatter profiles (S1 Fig panel B). ImageStreamX analysis of the morphology of cells within the monocyte, intermediate, and YS gates is shown in S1 Fig panel C. Cells in the monocyte gate displayed a predictable monocyte-like morphology, whereas cells within the intermediate gate displayed a more ‘activated’ and polarised morphology with numerous cytoplasmic granules and dendritic membranous extensions. Both morphologies were distinct from those of the YS macrophages. Quantitative analysis indicated that whilst there were no significant differences in the size of the monocyte or yolk-sac–derived populations between WT and ACKR2–/–mice (Fig 1Di), consistent and highly significant differences in the size of the intermediate population were seen in all experiments (Fig 1Dii). Further analysis revealed that the reduction in the size of the intermediate population in ACKR2–/–mice was also apparent in E14.5 skin, whilst by E17.5, this population was undetectable in the skins of WT and ACKR2–/–mice (Fig 1E).

Analysis of embryonic lungs at E14.5 indicated (S1 Fig panel Di, green arrow) that the intermediate cellular population was essentially absent from this tissue and remained absent throughout embryological development. Furthermore, there were no significant differences in the size of the monocyte or yolk-sac–derived populations in WT and ACKR2–/–lungs (S1 Fig panel Dii) at this time point. Similarly, the intermediate population was absent from foetal livers (S1 Fig panel 1Ei), and, again, no differences were seen in the size of the macrophage/monocyte populations in this tissue between WT and ACKR2–/–mice (S1 Fig panel Eii).

Thus, ACKR2–/–embryonic skin is characterised by a consistent and selective reduction in the size of an intermediate population of monocyte/macrophage-like cells.

### The intermediate population is transcriptionally distinct from monocyte- and yolk-sac–derived monocyte/macrophage populations

**Fig 2:**
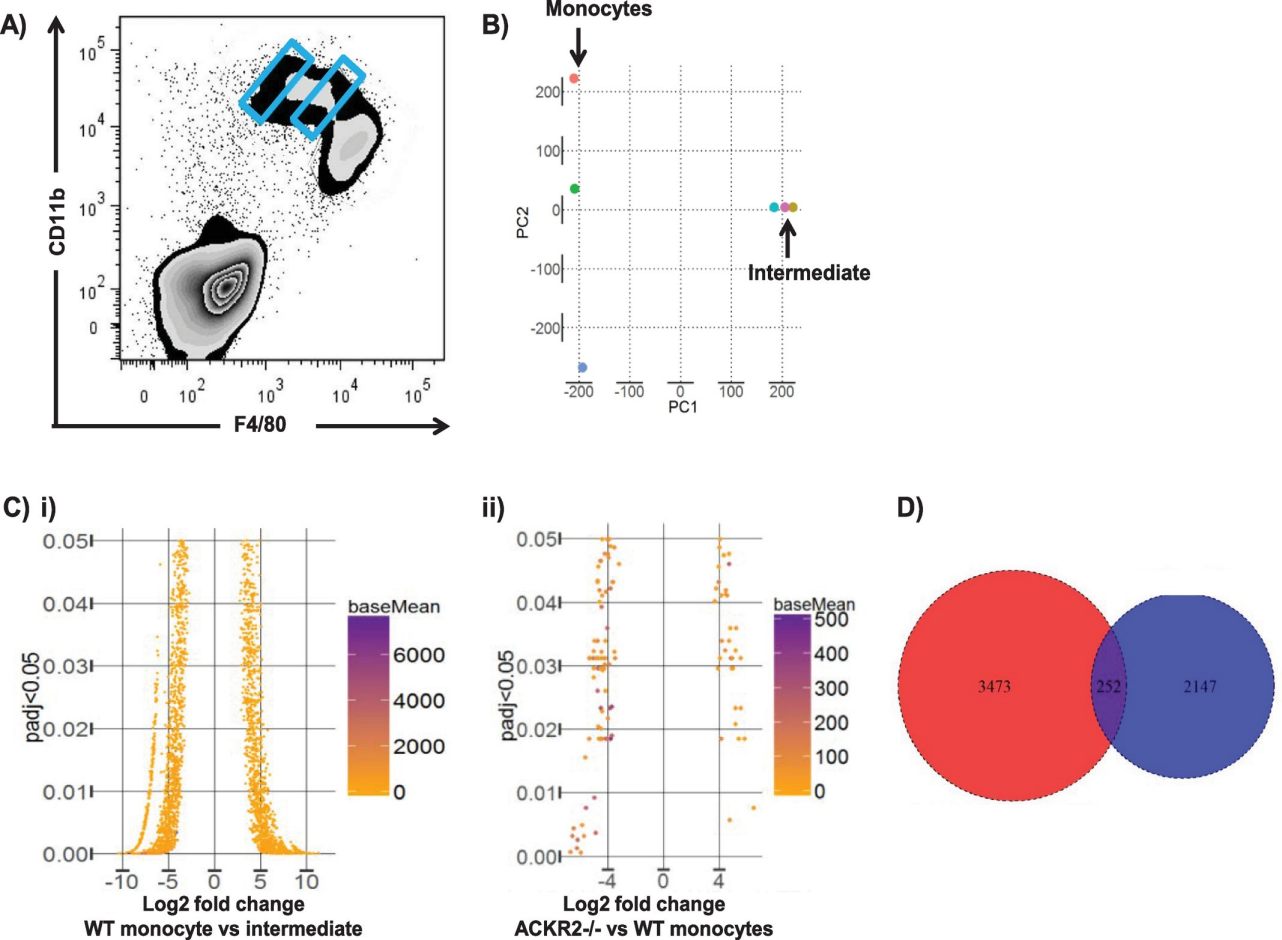
The intermediate cellular population is transcriptionally distinct from other embryonic monocyte/macrophage populations. A) The E14.5 monocyte and intermediate populations were purified by BD index sorting with gating on the extremes of the CD11bhi population (as marked in blue). B) Principal component analysis showing the lack of transcriptional relatedness of the monocyte and intermediate populations. C) Funnel plots showing the extent of i) transcriptional differences between the monocyte and intermediate populations in WT embryos and ii) transcriptional relatedness between the residual monocyte population in ACKR2–/–skin and the equivalent population in WT skin. D) Venn diagram showing limited overlap in the genes identified as being differentially expressed between monocyte and intermediate cells in the current study (blue circle) and those identified as being differentially expressed between YS and bulk monocytic cells in a previous study (red circle) [14]. Data associated with this figure can be found in the supplemental data files S1 Data and S2 Data. ACKR, atypical chemokine receptor; BD, Becton Dickinson; E, embryonic day; padj, adjusted *p* value; WT, wild type; YS, yolk sac.

Next, we carried out bulk RNA sequencing analysis to determine the transcriptional relatedness of the intermediate and monocyte-derived populations. These distinct populations were purified by gating on the extremes of the WT CD11bhi gate (blue gates in Fig 2A), RNA prepared and amplified prior to RNA sequencing (data accession number: PRJEB23797). Principal component analysis indicated that the two populations were transcriptionally distinct, although there was significant variability in the transcriptional profile of the sorted monocyte-derived cells (Fig 2B). Bioinformatic analysis revealed (Fig 2Ci) that there were 2,573 significantly differentially expressed genes between the monocyte-derived and intermediate cellular populations (1,224 increased and 1,349 decreased), indicating that these represent molecularly distinct cell types. Importantly, very few transcriptional differences were observed between the monocyte-derived population in WT skin and the remaining monocyte-derived population in ACKR2–/–skin (Fig 2Cii), and most of these differences related to minor contamination with residual intermediate population cells. These data indicate that the remaining monocyte-derived population in ACKR2–/–embryos is essentially identical to WT monocyte-derived cells, further confirming the loss of a discrete cellular population in the ACKR2–/–embryos. In addition, analysis of the differential gene expression patterns reported previously between monocyte and yolk-sac–derived cells in embryonic skin [14] revealed little overlap with the profile of differentially expressed genes in the current study (Fig 2D). Together, these transcriptomic data indicate that the intermediate population represents a transcriptionally distinct and previously uncharacterised embryonic monocyte/macrophage population.

Gene ontology analysis of differentially expressed genes revealed a preponderance of genes indicative of mature macrophage function in the intermediate cellular population (S2 Fig), and comparison with data on the Immgen site (www.immgen.org) again revealed transcriptional similarity to macrophages. These cells are characterised by high levels of expression of a range of gene families involved in phagocytosis and intracellular vesicle trafficking, as well as vacuolar ATPases and a number of other transcripts indicative of macrophage function. In addition, this population displays a very high level of expression of ribosomal proteins, as well as genes encoding other proteins involved in active translation. The intermediate population therefore appears to be highly translationally active and equipped for efficient phagocytosis and vesicle trafficking. A full list of differentially expressed genes is provided in S1 Data.

**Fig 3:**
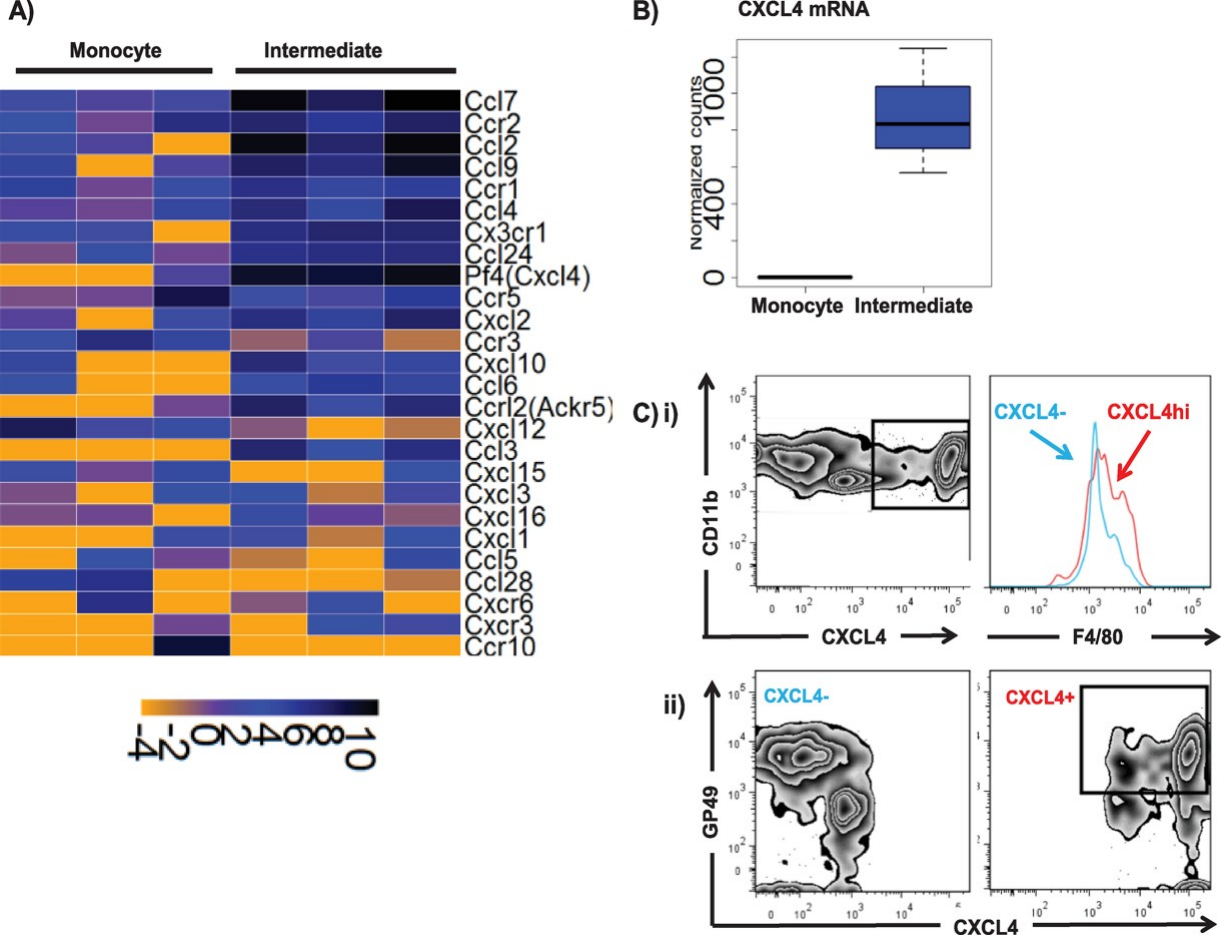
The intermediate cellular population expresses high levels of chemokines and their receptors. A) Heat map showing elevated expression of inflammatory chemokines and their receptors in the intermediate population compared to the monocyte population. B) CXCL4 is much more strongly expressed in intermediate population than in the monocyte population. C) i) Flow cytometric analysis demonstrating separate populations of CXCL4– and CXCL4hi cells within the overall CD11bhi gate (left-hand panel) and showing the CXCL4hi cells to be predominantly F4/80mid (right-hand panel; blue line, CXCL4– and red line, CXCL4hi). ii) Flow cytometric analysis demonstrating GP49 expression by CXCL4– and CXCL4hi myelomonocytic cells. Data associated with this figure can be found in the supplemental data file (S2 Data). ACKR, atypical chemokine receptor; CCL, CC ligand; CCR, CC chemokine receptor; CXCL, CXC ligand; CXCR, CXC chemokine receptor; GP49, glycoprotein 49; Pf4, platelet factor 4.

In addition, a large cohort of inflammatory chemokine and chemokine receptor genes is preferentially expressed in the intermediate population of cells (Fig 3A). The most striking difference in chemokine expression observed was the strongly increased expression of CXC ligand 4 (CXCL4) in intermediate population cells compared to monocyte-derived cells (Fig 3B). Flow cytometric analysis of the full CD11b gate (i.e., incorporating both the monocyte and intermediate populations) in CXCL4-reporter mice [20, 21] demonstrated that it is composed of distinct populations of CXCL4– and CXCL4+ cells (note that these cre recombinase (cre)-based mice simply report the presence or absence of CXCL4 promoter activity and not levels of expression), with the CXCL4+ cells displaying a predominantly F4/80mid phenotype (Fig 3Ci). Transcripts for the cell-surface marker glycoprotein 49 (GP49) (leukocyte-immunoglobulin–like receptor B4 [LILRB4]) were also strongly expressed in the intermediate population. Whilst the CXCL4– cells displayed mixed levels of GP49 expression, CXCL4hi cells were predominantly GP49+ (Fig 3Cii). Thus, bioinformatic analysis of the genes differentially expressed in the intermediate population of embryonic cells identifies them as a novel, to our knowledge, population of CXCL4hi GP49+ macrophage-like cells. We are in the process of attempting to characterise these cells in more detail, and the remainder of the present study is specifically focused on analysing mechanism behind the disruption of this cellular population in ACKR2–/–embryos.

### ACKR2 interacts with the CCR2/CCL2 axis to regulate intermediate cell recruitment to embryonic skin

**Fig 4:**
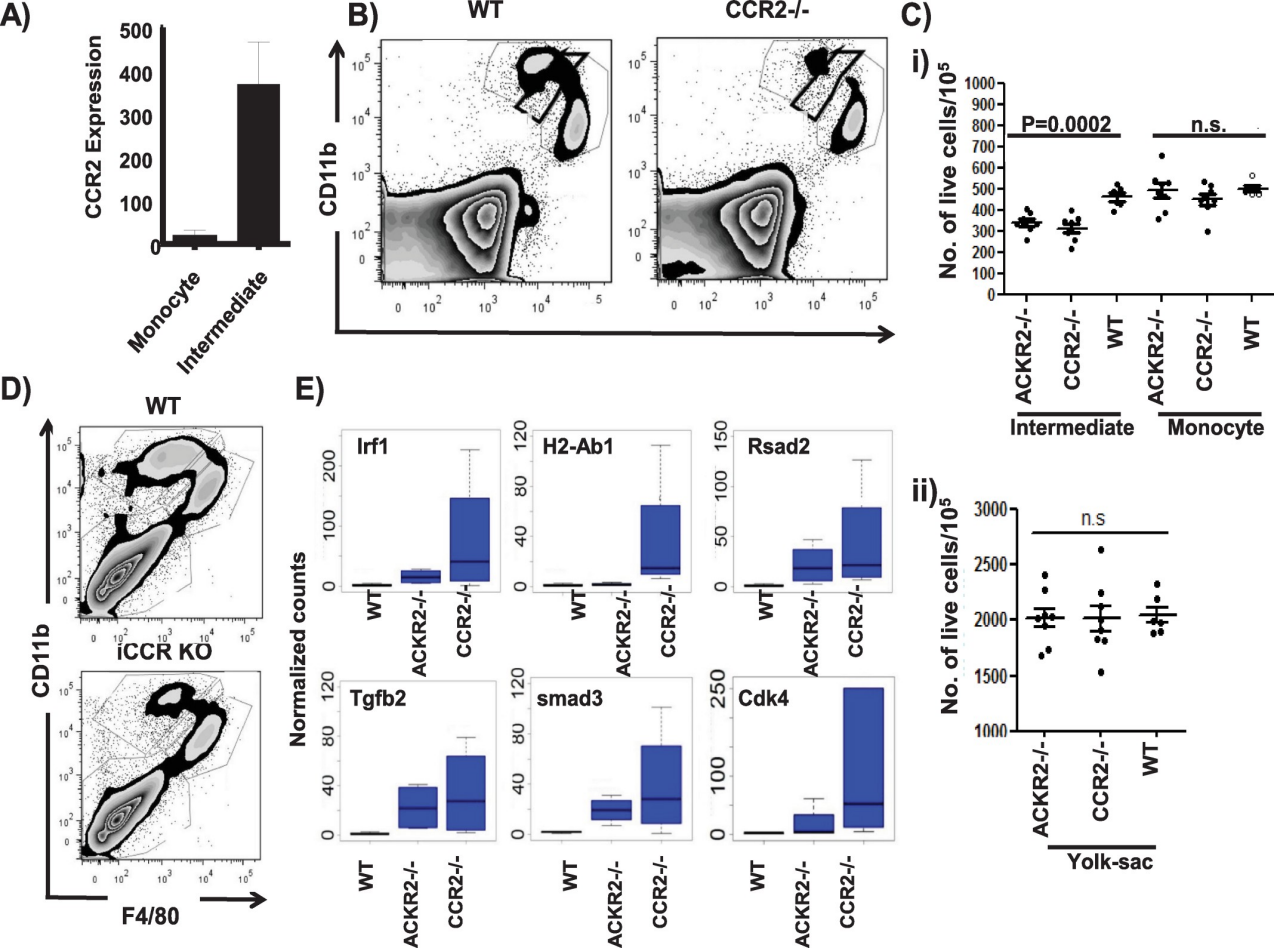
CCR2–/–embryos phenocopy ACKR2–/–embryos. A) CCR2 expression is higher in the intermediate compared to the monocyte population of cells. These data represent raw counts of CCR2 expression from the bulk RNA sequencing data. B) Flow cytometric analysis of CD11b and F4/80 expression in CD45+ cells from skins of E15.5 WT and CCR2–/–embryos. C) Quantification of multiple flow cytometric analyses of the monocyte, intermediate, and YS populations in skin from E15.5 WT, CCR2–/–, and ACKR2–/–embryos. Data shown here are from 1 of 2 repeated experiments, and Mann–Whitney U test was used to test for significance. D) Flow cytometric analysis of CD11b and F4/80 expression in CD45+ cells from skins of E15.5 WT and iCCR–/–embryos. E) Relative expression (normalised counts of DESeq2 outputs) of genes preferentially expressed in the monocyte population from WT (*n* = 3 embryos), ACKR2–/–and CCR2–/–E14.5 embryonic skin (*n* = 4 embryos). Data associated with this figure can be found in the supplemental data file (S2 Data). ACKR, atypical chemokine receptor; CCR, CC chemokine receptor; Cdk4, cyclin-dependent kinase 4; DESeq2, differential expression sequencing 2; E, embryonic day; iCCR, inflammatory CC chemokine receptor; KO, knockout; lrf1, liver regeneration factor 1; n.s., not significant; Rsad2, Radical S-Adenosyl-Methionine-Domain–Containing 2; smad3, smad family member 3; Tgfb2, transforming growth factor beta 2; WT, wild type; YS, yolk sac.

In terms of chemokine receptors, monocyte/macrophage receptor CCR2 is expressed at significantly higher levels in intermediate population cells compared to monocyte-derived cells (Fig 3A and Fig 4A). Interestingly, embryonic skin from CCR2–/–mice displayed a similar phenotype to that observed in ACKR2–/–embryonic skin with a specific and significant reduction in the size of the intermediate population (Fig 4B and 4C). We recently generated mice with a compound deletion in other receptors involved in inflammatory monocyte migration [22]. These mice are deficient in CCR1, CCR2, CCR3, and CCR5, and analysis of embryonic skin monocyte/macrophage populations in these mice (inflammatory CC chemokine receptor [iCCR]–/–mice) revealed an essentially identical phenotype to that observed with CCR2–/–mice (Fig 4D). Therefore, there appears to be no redundant, compensatory, or additive roles of CCR1, CCR3, or CCR5 in the phenotype observed. The selective depletion of the intermediate population in CCR2–/–mouse skin was again confirmed by RNA sequencing, which showed that the residual monocyte-derived population in CCR2–/–mice displayed an essentially identical transcriptional profile to the equivalent population in ACKR2–/–mice, with only 14 significant gene differences being detected. Notably (sample data provided in Fig 4E), alterations in expression levels of many of the genes associated with the intermediate population are more marked in the residual monocyte population in CCR2–/–embryos, indicating more comprehensive depletion of the intermediate cellular population compared with ACKR2–/–embryos. Overall, these data show that CCR2–/–embryos present as a phenocopy of ACKR2–/–embryos and that the intermediate population therefore requires CCR2 for aspects of migration to embryonic skin.

### CCL2 expression in the developing embryo

**Fig 5:**
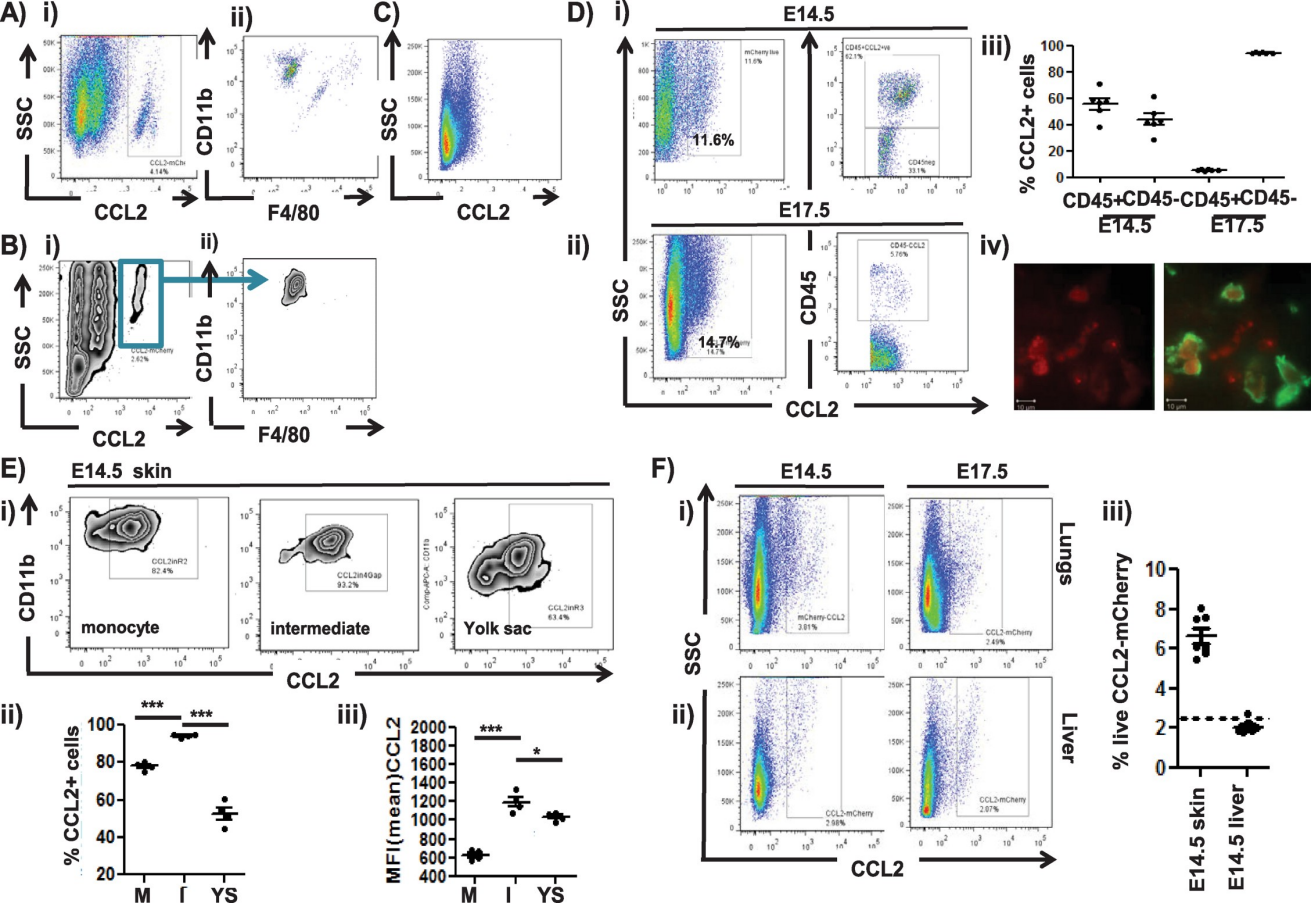
Analysis of CCL2 expression in embryonic tissues. A) Flow cytometric analysis of E12.5 total YS cells from CCL2-reporter mice showing i) a discrete CCL2-expressing population and ii) CD11b and F4/80 gating, demonstrating that this population is largely CD11bhiF4/80lo. B) Flow cytometric analysis of E12.5 total skin cells from CCL2-reporter mice showing i) a discrete CCL2-expressing population and ii) CD11b and F4/80 gating, demonstrating that this population is CD11bhiF4/80lo. C) Flow cytometric analysis of E12.5 total foetal liver cells from CCL2-reporter mice indicating the absence of CCL2 expression. Data are representative of 3 repeat experiments. D) Flow cytometric analysis of CCL2-expressing cells from i) E14.5 and ii) E17.5 skin from CCL2-reporter mice. The left-hand panels show total cell CCL2 expression, and the right-hand panels assign expression to CD45+ or–cells. iii) Quantification of CCL2 expression amongst CD45+ and–cells from E14.5 and E17.5 embryonic skin. iv) Dual Lyve-1 (Green) staining showing CCL2 (red) expression by skin resident macrophage-like cells in E15.5 skin whole mounts. E) i) Flow cytometric analysis of CCL2 expression in the distinct E14.5 monocyte, intermediate, and YS populations. Quantification as ii) percentage of live CCL2+ cells and iii) MFI of CCL2 expression amongst the monocyte (M), intermediate (I), and YS populations. One-way ANOVA with Bonferroni post-test for comparisons between two groups. F) Flow cytometric analysis of CCL2 expression in i) lungs and ii) livers from E14.5 and E17.5 embryos. iii) Comparative quantification of CCL2 expression in E14.5 skin and liver (dotted line = background level from nonreporter foetal tissues). Data associated with this figure can be found in the supplemental data file (S2 Data). CCL, CC ligand; E, embryonic day; Lyve-1, Lymphatic vessel endothelial hyaluronan receptor 1; MFI, mean fluorescent intensity; SSC, side scatter; YS, yolk sac.

To gain insights into the homing of the intermediate cells within the embryo, we next used CC ligand 2 (CCL2)-reporter mice [23] to examine expression of the CCR2 ligand, CCL2, in embryonic tissues. The earliest detection of CCL2 was at E12.5 in YS and skin (Fig 5A and 5B). Notably, in both tissues, CCL2 expression was predominantly confined to a population of CD11bhiF4/80lo monocyte-like cells. In repeated experiments, we never observed CCL2 expression in E12.5 skin without coincident expression in the YS, but we occasionally observed expression in YS monocytes without expression in skin (sample data shown in S1 Table). Together, these data suggest that monocytic cells express CCL2 early in embryogenesis and that they appear in the YS just prior to their appearance in the skin. Notably, CCL2-expressing cells were absent from foetal livers at E12.5 (Fig 5C).

In E14.5 skin (Fig 5Di), CCL2 was equally expressed by both leukocytes (CD45+) and nonleukocytes (CD45–). In contrast (Fig 5Dii), at E17.5, skin CCL2 was almost entirely expressed by nonleukocytic cells (Fig 5Diii). Further imaging of CCL2 expression in embryonic skin at E14.5 confirmed that the leukocyte source of this chemokine was Lymphatic vessel endothelial hyaluronan receptor 1 (Lyve-1)+ cells that do not display a lymphatic vessel phenotype and thus are likely to be Lyve-1+ macrophages (Fig 5Div) and that the nonleukocyte source was neither blood nor lymphatic endothelium and may therefore be either keratinocytes or fibroblasts. More detailed analysis of CCL2 expression in E14.5 embryonic skin monocytes/macrophages revealed expression by cells within each of the monocyte, yolk-sac–derived, and intermediate population gates, with moderately higher expression levels being seen in the intermediate population (Fig 5E). In contrast to the skin, CCL2 expression was only weakly detectable in lungs and, again, virtually undetectable in foetal livers at either E14.5 or E17.5 (Fig 5F).

Thus, CCL2 is expressed by both monocyte/macrophage cells and stromal cells in the developing skin and is therefore likely to contribute to the homing of the intermediate population to this embryonic site.

### Placental ACKR2 supports intermediate cell migration in the embryo by blocking maternal chemokine entry into the embryonic circulation

**Fig 6:**
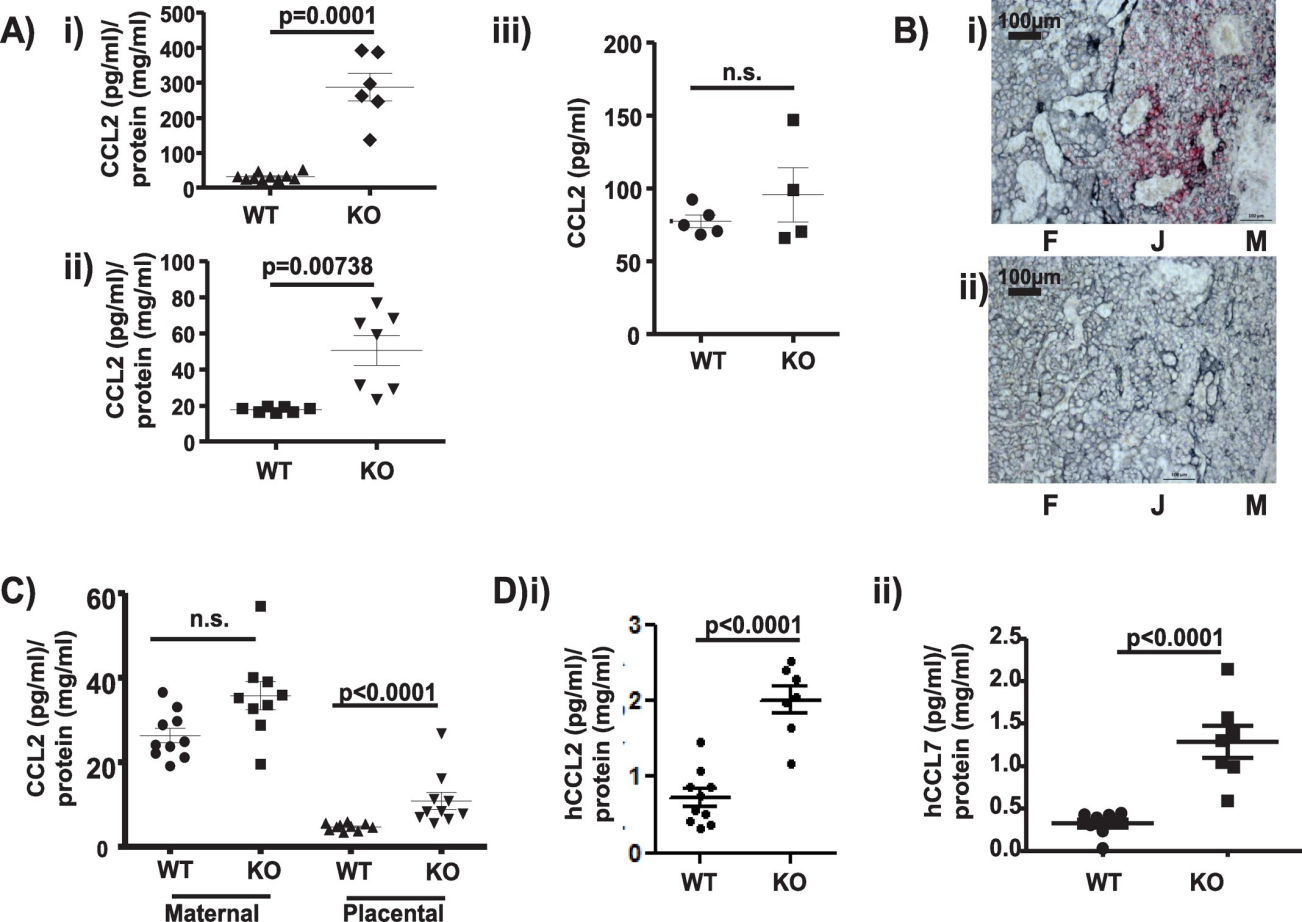
Placental ACKR2 limits chemokine movement from the maternal decidua to the embryo. A) MSD-based analysis of CCL2 concentrations (normalised to total protein concentration) in plasma from i) E14.5 and ii) E15.5 WT and ACKR2–/–embryos. iii) CCL2 levels in adult pregnant mouse plasma expressed as pg/ml. Data shown here were derived from 1 of 2 repeated experiments. Unpaired Student *t* test without (i) and with Welch’s correction (ii) was used for statistical comparison between groups. B) In situ hybridisation showing (in red) i) ACKR2 expression (red coloration) in trophoblastic cells (grey coloration) in the E14.5 WT murine placenta and ii) lack of expression in ACKR2–/–placenta. F = foetal side of the placenta, J = foetal/maternal junction, and M = maternal decidua. Scale bar = 100 μm. C) MSD analysis of CCL2 levels (normalised to total protein concentration) in the maternal decidua and placenta of WT and ACKR2–/–E14.5 embryos. Data shown here are from 1 experiment. Similar results were independently obtained with E13.5 placenta lysates. D) Quantification of i) human CCL2 and ii) human CCL7 levels (normalised to total protein concentration) in plasma of embryos from mothers, systemically, injected i.v. with 500 ng of each chemokine. Data shown here are from 1 experiment. Unpaired Student *t* test without Welch’s correction was used for statistical comparison of injected human CCL2 (i) between genotypes. Data associated with this figure can be found in the supplemental data file (S2 Data). ACKR, atypical chemokine receptor; CCL, CC ligand; E, embryonic day; hCCL, human CCL; i.v., intravenously; KO, knockout; MSD, mesoscale discovery; n.s., not significant; WT, wild type.

Together, the above data suggest that early monocyte-derived, and potentially later stromal-derived, CCL2 is involved in recruitment of the intermediate population to embryonic skin and that aspects of this recruitment process are dependent on both CCR2 and ACKR2. One possibility, therefore, is that the CCR2 ligand gradient between the circulation and the skin is disrupted in ACKR2–/–embryos because of lack of active scavenging. To test this, we measured CCL2 concentrations in the plasma of WT and ACKR2–/–embryos, and as shown in Fig 6A, E14.5 (i) and E15.5 (ii) ACKR2–/–plasma displayed significantly higher CCL2 levels compared to WT plasma. Indeed, CCL2 was essentially undetectable in the plasma of WT embryos. No differences were detected in the plasma CCL2 concentrations in adult pregnant WT or ACKR2–/–mice (Fig 6Aiii). These data suggest that ACKR2 actively regulates CCL2 concentrations in embryonic plasma, thus maintaining gradients between the circulation and peripheral tissues. In humans, a very prominent site of strong ACKR2 expression is the placenta, where it is specifically localised to trophoblasts [24, 25]. Similarly, in the mouse, using in situ hybridisation (there are currently no antibodies capable of detecting murine ACKR2), we detected widespread expression of ACKR2 in trophoblastic cells in the placenta (Fig 6B). Colocalisation of ACKR2 and the trophoblastic marker cytokeratin 7 is shown at higher magnification in S3 Fig, panel A. Importantly, no significant domains of ACKR2 expression were detected in embryos, suggesting that intraembryonic ACKR2 is unlikely to contribute to the regulation of embryonic plasma CCL2 (S3 Fig panel B). In addition, flow cytometric analysis using Alexa-Fluor labelled CCL22 (an ACKR2 ligand) demonstrated the presence of CCL22-binding cells in the WT placenta but not decidua, which were absent in both placenta and decidua from ACKR2–/–mice (S3 Fig panel C). These cells are phenotypically characterised as being CD45–/CD90– and are therefore nonleukocyte and nonfibroblastic. These cells express stem cell antigen 1 (Sca1), which is a marker for murine trophoblasts [26], and thus appear to be trophoblastic in nature.

We detected strong expression of the CCR2 ligand CCL2 in the maternal decidua (Fig 6C) in both WT and ACKR2–/–mice, where it is likely to be involved in local recruitment of decidual macrophages and natural killer (NK) cells [27]. In contrast, only very low levels of CCL2 were detected in the placenta proper in WT mice, whereas it was easily detectable in ACKR2–/–mouse placenta. In support of these observations, whilst we observed no correlation between CCL2 concentrations in the maternal decidua and the placenta in WT mice, we observed a very strong correlation between levels in these two compartments in ACKR2–/–mice (S4 Fig, panel A). Interestingly, this elevation of CCL2 within the placenta does not alter the macrophage content of either the maternal decidua or placenta (S4 Fig, panel B). Overall, these data strongly indicate a key role for the trophoblast layer, between the decidua and the foetus, in restricting chemokine movement from the mother to the foetal circulation.

We next examined the potential source of CCL2 within the maternal decidua. PCR analysis (S4 Fig panel C) demonstrated that in contrast to in vitro macrophages, CCL2 transcripts were essentially undetectable in the maternal decidua or placenta of either WT or ACKR2–/–embryos. This suggests that the ultimate source of the CCL2 that enters the foetal circulation is likely to be maternal blood within the decidua.

Overall, therefore, we reasoned that ACKR2 was potentially compartmentalising CCR2 ligand function within the maternal decidua and thus preventing it from interfering with CCR2-dependent monocyte/macrophage cell migration within the embryo. To test this, we intravenously injected 2 human CCR2 ligands (hCCL2 and hCCL7) into pregnant WT and ACKR2–/–mice and measured their levels in embryonic plasma to gauge the effectiveness of ACKR2 in preventing their movement from the maternal decidua to the embryonic circulation. Importantly, the assay used did not detect any CCL2 or CCL7 in the plasma of embryos from mock-injected mothers, confirming the species specificity of the detection methodology. As shown in Fig 6D, both (i) hCCL2 and (ii) hCCL7 were detected at considerably higher levels in the plasma of ACKR2–/–compared to WT embryos. These experiments confirm a role for ACKR2 in compartmentalising CCR2 ligand function within the maternal decidua and provide a mechanistic explanation for the developmental defect in macrophage development in ACKR2–/–embryos.

## Discussion

Chemokines are fundamental regulators of in vivo leukocyte migration [1]. Here, we demonstrate a novel, to our knowledge, role for the chemokine receptor CCR2 in regulating the intraembryonic migration and localisation of a novel, to our knowledge, and currently uncharacterised population of macrophages. Importantly, we also demonstrate that the integrity of this process is crucially dependent on the expression of the atypical chemokine receptor ACKR2, which scavenges chemokines at the maternal/foetal interface and compartmentalises chemokine activity within the maternal decidua. This compartmentalisation mechanism ensures that maternally derived chemokines can perform localised functions without interfering with CCR2-dependent macrophage migratory dynamics in the embryo. This mechanism is summarised in S5 Fig panels A and B. These observations also explain the fact that ACKR2 and its scavenging activity are highly maintained and conserved amongst mammals. In contrast, its likely evolutionary precursor in premammalian species lacks the common DKYLEIV motif and may thus be involved in alternative aspects of chemokine biology [28]. Given that additional atypical chemokine receptors are also expressed in the placenta [29, 30], we propose that this model of chemokine compartmentalisation may be important for protecting other intraembryonic cellular migratory activities. This study, and its conclusions, represents a major departure from previous publications regarding roles for ACKR2 in the placenta [24], which have suggested it to be important only in the context of systemic maternal inflammation. The current study suggests that there is a more basic and homeostatic role for ACKR2 regardless of the maternal inflammatory status. The previous studies have indicated enhanced embryonic rejection in systemically inflamed ACKR2–/–mice, and, whilst this has been proposed to relate to inflammatory leukocytes in the placenta, our data would suggest that this may more likely relate to a profound breakdown in the chemokine compartmentalisation mechanism with attendant effects on embryonic development. It is likely that this novel, to our knowledge, mechanism is of even more marked importance in protecting the offspring of immunologically mismatched parents [31].

What is not clear from our study is exactly how chemokines are transferred from the maternal decidua to the foetal circulation. It is unlikely that the trophoblast layer is permeable to chemokines, and thus alternative options, such as transfer through pinocytosis [32], may present a plausible explanation. In this scenario, the presence of ACKR2 on the surface of the WT trophoblasts would ensure that any of its ligands associating with this cellular surface would be internalised and degraded. Accordingly, in the absence of ACKR2, chemokines may associate with the trophoblastic cell surface through interactions with glycosaminoglycans [33]. These glycosaminoglycan-bound chemokines could then be transferred to the foetal circulation by pinocytosis and released as a natural consequence of the chemokine on–off interaction with cell-surface glycosaminoglycans.

This potential mechanism may also explain the apparent disconnect between relatively low maternal peripheral blood CCL2 levels (see Fig 6Aiii) and the higher levels of CCL2 seen in ACKR2–/–foetal plasma (approximately 400 pg/ml). Specifically, this could be achieved by the effective concentration of chemokine within the foetal circulation by multiple rounds of pinocytosis of membrane-deposited maternal chemokines. It is worth noting that even at approximately 400 pg/ml, this represents a molarity (40 pM) that is considerably below the Kd of CCL2 binding to CCR2 (2.1 nM) [34]. This would explain why the reduction of the intermediate population in ACKR2–/–embryos is partial and not complete. In addition, however, other CCR2-binding chemokines such as CCL7 and CCL12 are also likely to enter the foetal circulation and thus further increase the overall plasma concentration of CCR2-binding chemokines.

This novel, to our knowledge, intermediate macrophage population is crucially dependent on CCR2 and maternal chemokine compartmentalisation by ACKR2 for migration and positioning in embryonic tissues, especially skin. Our analysis of the intraembryonic expression of the CCR2 ligand, CCL2, indicates that it is first detected in skin at E12.5, expressed by cells with a classic monocyte phenotype. We propose that these cells precede the intermediate macrophage population into the skin and serve as an early source of local cutaneous CCL2 to support the subsequent recruitment of this population into the skin (S5 Fig panel C). In ACKR2–/–embryos, the enhanced levels of maternally derived CCL2 in the embryonic circulation prevent appropriate migration of these cells. One further option might have been that the intermediate population simply represents CCR2-expressing macrophages differentiated from the monocyte population, which are then recruited away for the skin by the increased plasma CCL2 levels. However, the fact that these cells are also reduced in the skin of CCR2–/–embryos argues against this and further strengthens the argument that the monocyte and intermediate cell populations are distinct.

Overall, therefore, this study identifies CCR2 and its ligand CCL2 as being essential for effective intraembryonic migration of a novel, to our knowledge, population of intermediate phenotype macrophages. The data highlight ACKR2 as being indispensable for ensuring the robustness of this process by blocking movement of maternally derived chemokines into the embryonic circulation. We propose that this model of chemokine compartmentalisation accounts for the mammalian-specific nature of ACKR2 and provides a template for interpreting the activity of other placentally expressed atypical chemokine receptors in regulating maternal–foetal chemokine exchange.

## Materials and methods

### Ethics statement

All animal experimentation was approved by the local Ethical Review Board and by the UK Government Home Office licencing authorities and was performed under the auspices of a UK Home Office Project Licence.

### Animal maintenance

ACKR2-deficient mice were generated as reported [35] and backcrossed onto the C57BL6/J background for at least 10 generations. CCR2-deficient mice, CCL2-reporter mice [36], and MacGreen mice [37] were purchased from The Jackson Laboratory (Bar Harbor, ME, USA). Pf4iCre mice [21] were crossed with Rosa26-tdTomato mice [38] to generate Pf4 (CXCL4) reporter mice. All mice were maintained in the specific pathogen-free Animal Research Facility of The Beatson Cancer Research Institute.

### Harvesting embryonic tissues and foetal parts of placenta

Embryonic dorsal skin, livers, lungs, and YSs were dissected with fine spring bow scissors and forceps (Fine Science Tools, North Vancouver, BC, Canada), and maternal decidua and the placenta were isolated by dissection as described [39]. Embryonic skin and YSs were minced and digested in an HBSS (Gibco, Gaithersburg, MD, USA)-based enzymatic cocktail consisting of 1 mg/ml collagenase-D (Roche, Basel, Switzerland), 500 μg/ml dispase-II (Thermo Fisher Scientifc, Waltham, MA, USA), and 100 μg/ml DNase-I (Roche). Skin fragments were shaken at 37°C in enzymatic cocktail (1 ml per skin sample) for 1 hr at 1,000 rpm on a temperature-controlled shaker (Eppendorf, Hamburg, Germany). YS fragments were digested in the same enzymatic cocktail (1 ml volume per YS) at 37°C and shaken every 10 to 15 min. For digestion of embryonic livers, lungs, and foetal parts of the placenta, tissues were incubated in 1 ml HBSS-based digestion cocktails comprising 800 μg/ml dispase-II (Thermo Fisher Scientific), 200 μg/ml collagenase-P (Roche), and 100 μg/ml DNase-I (Roche) with shaking at 37°C at 400 rpm on a temperature-controlled shaker (Eppendorf) with occasional inverting by hand. 1 ml of RPMI/10% serum/100 U/μg/ml penicillin/streptomycin (Invitrogen, Carlsbad, CA, USA; Thermo Fisher Scientific)/10 μg/ml Gentamycin (Sigma-Aldrich, St. Louis, MO, USA)/10 mM HEPES (Sigma-Aldrich) was added to each 1 ml of tissue digest, mixed, and then filtered through 70-μm nylon cell strainers (Grenier, Stonehouse, Gloucestershire, UK). Filtered tissue digests were centrifuged and cell pellets washed twice in 1 ml FACS buffer (PBS/0.1% BSA/0.1% NaN_3_/2 mM EDTA).

### Collection of maternal and embryonic plasma

A mixture of 500 ng of hCCL2 (Cat no. 2798-MC-050/CF; R&D Systems, Minneapolis, MN, USA) and 500 ng of hCCL7 (Cat no. 282-P3-010; R&D Systems) was prepared in sterile filtered PBS in a total volume of 110 μl (1.5 ml Eppendorf tube) and injected i.v. into pregnant ACKR2-deficient or WT females (E15.5). One hr later, blood was collected from the injected pregnant female by venipuncture into a 1 ml syringe filled with 50 μl of filtered 0.2 M EDTA/PBS and then left at 4°C in a 1.5 ml Eppendorf tube. Embryos were rinsed twice in separate dishes of PBS to avoid contamination from maternal blood and then immediately decapitated, and blood was allowed to drip (at 4°C) to the bottom of 1.5 ml Eppendorf tubes filled with 20 μl of 0.2 M EDTA. The blood was then centrifuged at 2,000 × *g* for 5 min at 4°C to collect the plasma, which was stored at –80°C until use.

### Chemokine measurements

Adult plasma was diluted 2-fold into a final volume of 50 μl and levels of murine CCL2 measured using an MSD Mouse MCP-1 Ultra-Sensitive Kit (Meso Scale Discovery, Rockville, MD, USA). For human CCL2 and CCL7 measurements, plasma was diluted 2.5-fold into a final volume of 50 μl and chemokine levels measured using an MSD U-PLEX kit (Meso Scale Discovery, https://www.mesoscale.com/en/products_and_services/assay_kits/u-plex_gateway/), according to the manufacturer’s instructions. Placenta was separated into maternal decidua and foetal components, each of which were minced in 200 μl of 1× PathScan Sandwich ELISA Lysis Buffer (1×) (Cell Signaling, Danvers, MA, USA) supplemented with 1 mM PMSF. These samples were mixed overnight at 4°C by rotation, and the supernatants were harvested by centrifugation at 14,000 × *g* for 2 mins and stored at −80°C until use. Protein concentrations in the plasma and placental lysates were measured using a Pierce BCA Protein Assay Kit (Thermo Fisher Scientific) for subsequent normalisation of chemokine levels. Note that adult plasma chemokine concentrations are simply expressed as pg/ml, whereas chemokine concentrations in foetal blood are expressed as a function of total protein concentration. This is because, whilst we can measure concentrations in clearly defined volumes of adult plasma, the fact that foetal plasma was collected into PBS and therefore the volume of plasma collected was not defined meant that normalisation in terms of protein concentration was deemed the most appropriate way to express these data.

### Antibodies

Suppliers/sources of antibodies used for this study are provided in S2 Table.

### Flow cytometry

Each tissue digest was firstly stained with 0.5 μg of FcBlock on ice for 20 min and then stained with the antibody panels (on ice for 30 min) listed in the relevant figure legends. For dead cell exclusion, cells were stained in Fixable Viability Dye eFluor 780 dye (Thermo Fisher Scientific) diluted 1:1,000 in a volume of 400 μl to 500 μl PBS/2 mM EDTA. Fresh stained cells from embryonic digests were resuspended in a volume of 200 μl to 400 μl PBS/2 mM EDTA and analysed on a BD LSRII flow cytometer using the CST setting (BD Bioscience, San Jose, CA, USA). In some FACS experiments, cell suspensions were run on a BD Fortessa flow cytometer (BD Bioscience). For placenta cell digests, stained cells were fixed in 4% PFA and then acquired the next day on a BD LSRII flow cytometer. A total of 1 × 10^5^ to 2 × 10^5^ events were acquired with dead cells, and cell doublets were excluded from data analysis. All flow cytometry data were analysed using FlowJo software.

For staining of CD31 (Meca13.3) and Lyve-1, biotin-conjugated Meca13.3 (Biolegend, San Diego, CA, USA) and goat anti-mouse Lyve1 antibodies (R&D Systems) were used. A cocktail of 1 μg/ml strepavidin-PerCP and 4 μg/ml chicken anti-goat IgG (H + L) with 50 μl each was added to each skin digest. All staining was done in round-bottomed Falcon tubes (BD Bioscience), apart from placental digests, which were incubated with a combination of 0.5 μg of FcBlock and antibody cocktails together in sterile round-bottomed tissue culture 96-well plates (Corning, Corning, NY, USA) in a volume of 200 μl.

### Whole-mount immunolabelling of CCL2 in the murine embryonic skin at E15.5

CCL2-reporter and WT embryos were fixed in ice-cold 1% PFA/PBS for 48 hrs at 4°C and then left in PBS at 4°C until use within 2 months. 250 μl of antibody cocktail made up of 4 μg/ml polyclonal rabbit anti-mCherry antibody (Abcam, Cambridge, UK) + 2 μg/ml of polyclonal goat anti-Lyve1 Ab (R&D Systems) in filtered TBS/1% fish gelatine (Sigma-Aldrich)/0.05% Triton X-100 was prepared. A volume of 30 μl antibody cocktail was then added to each well of a round-bottomed 96-well tissue culture plate with a piece of dorsal skin sheet and the plate gently shaken O/N at 4°C. Next day, stained skin sheets were washed ×3 at RT in filtered TBS/1% fish gelatine for 5 min each. Stained skin sheets were labelled with 3 μg/ml of chicken anti-goat-IgG and 3 μg/ml of chicken anti-rabbit-IgG in a final volume of 50 μl of TBS/1% fish gelatine for 1 hr at RT. This was followed by ×3 wash at RT for 5 min each and then fixation for 5 min in 4% PFA at RT before washing ×3 in PBS for 5 min each. Each stained skin sheet was left in 20 μl of PBS in wells of a 96-well round-bottomed tissue culture plate until imaging on the Zeiss ELYRA microscope (Zeiss, Oberkochen, Germany) the next day. Stained dorsal skin sheets were placed into a 35-mm glass-bottomed dish (MatTek Corporation, Ashland, MA, USA) and imaged on a ×63 lens (Plan-Apochromat 63×/1.4 Oil DIC M27, Zeiss) with immersion oil (Zeiss). Raw images were captured and saved as czi images on Zeiss ZEN Black and then exported as tif images.

### Statistical analysis

Comparison between groups was performed using either unpaired Student *t* test or Mann–Whitney test, depending upon the normal distribution probability checked for by either Kolmogorov–Smirnov test or Shapiro test on Prism5 or R (×64 3.2.1). Welch correction was applied to unpaired Student *t* test if variances between groups were significant *p* < 0.05. Correlation analysis was done using stat functions provided on the R documentation (https://stat.ethz.ch/R-manual/R-devel/library/stats/html/cor.test.html). All error bars are SEM, and each dot on dot plots represents a single mouse or embryo. All data with *p* < 0.05 were considered to be significant.

Additional Materials and Methods details are provided in S1 Text.

## Supporting information

S1 Fig Further analysis of the ‘intermediate’ population of cells. A) Flow cytometry profile showing the previously characterised populations of monocyte-derived (CD11bhiF4/80lo; red arrow) and yolk-sac–derived (CD11bloF4/80hi; green arrow) myelomonocytic cells in E145 embryonic skin. B) Flow cytometric analysis of the SSC/FSC profile of the intermediate cellular population. C) ImageStreamX analysis of the monocyte, intermediate, and YS populations showing the pregating strategy and exemplar morphology images. D) i) Flow cytometric analysis of CD11b/F4/80 expression by CD45+ myelomonocytic cells in embryonic lung at E14.5. ii) Quantitative analysis of monocyte and yolk-sac–derived cell numbers from multiple flow cytometric analyses of E14.5 WT and ACKR2–/–lungs from one experiment. E) i) Flow cytometric analysis of CD11b/F4/80 expression by CD45+ myelomonocytic cells in foetal liver at E14.5. ii) Quantitative analysis of monocyte (CD45+CD11b+F4/80–) and yolk-sac (CD45+CD11b+F4/80+)–derived cell numbers from multiple analyses of E14.5 WT and ACKR2–/–livers. ACKR, atypical chemokine receptor; E, embryonic day; FSC, forward scatter; SSC, side scatter; WT, wild type; YS, yolk sac.
(EPS)

S2 Fig Heatmap analysis of genes differentially expressed between the monocyte and intermediate cellular populations. Genes involved in A) phagocytosis and intracellular vesicle trafficking, B) vacuolar ATPases, C) macrophage function, and D) translation. Horizontal bar plots (A) showing the log2 fold change values on A) and B) were drawn using Python matplotlib with *y*-axis representing log2 fold change (DESeq2) of intermediate cells versus monocytes. Numbers in the boxed areas are transformed values of the gene counts. Data associated with this figure can be found in the supplemental data file (S1 Data). DESeq2, differential expression sequencing 2.
(EPS)

S3 Fig RNAscope in situ hybridisation analysis of ACKR2 expression in E13.5 embryos. A) higher magnification representation of a portion of Fig 6Bi showing ACKR2 expression as detected by in situ hybridisation (red) and colocalisation with cytokeratin staining (black/grey). B) Low-magnification imaging of in situ hybridisation analysis showing lack of ACKR2 expression in the left-hand panels of i) WT and ii) ACKR2–/–embryos. Right-hand panels represent positive in situ hybridisation staining for PPIB. C) summary results from flow cytometric analysis of cells binding the ACKR2 ligand CCL22 in the maternal decidua (M) and placenta (P) of WT and ACKR2–/–embryos. These cells are negative for CD45 and are therefore not leukocytes. In addition, they are positive for Sca1 and negative for CD90, suggesting that they are nonfibroblastic and likely trophoblastic in nature. ACKR, atypical chemokine receptor; CCL, CC ligand; E, embryonic day; PPIB, peptidyl isomerase B; Sca1, stem cell antigen 1; WT, wild type.
(EPS)

S4 Fig Analysis of CCL2 and macrophage levels in WT and ACKR2–/–decidual and placental tissues. A) Graph showing lack of correlation between placental and decidual CCL2 in WT mice but strong correlation in ACKR2–/–mice. B) Summary of flow cytometric analysis of macrophage (CD11b+F480Lo) numbers as percentage of total CD45+ cells in WT and ACKR2–/–decidua and placenta. No significant differences were seen between any of the groups. C) Results of PCR analysis of CCR2 expression in maternal decidua and placenta from WT and ACKR2–/–embryos. Data on expression in macrophages are included as a positive control. ACKR, atypical chemokine receptor; CCR, CC chemokine receptor; WT, wild type.
(EPS)

S5 Fig A model of chemokine compartmentalisation. A) In WT placenta, i) ACKR2 prevents CCL2 movement from the maternal decidua into the embryonic circulation, thereby facilitating ii) the localised recruitment of CCR2-expressing cells to sources of CCL2 in embryonic skin. B) In ACKR2–/–placenta, i) there is no barrier to the influx of maternal CCL2 into the foetal circulation, and this leads to a disruption of the CCL2 gradient between the circulation and the skin in the embryos. C) A model for the stepwise recruitment of intermediate cells to embryonic skin. The earliest CCL2-expressing cells in embryonic skin are classical monocytic cells, and these precede the intermediate cellular population. The CCL2 produced by the monocytic population recruits CCR2+ intermediate cells. These cells colonise the skin by E14.5. ACKR, atypical chemokine receptor; CCL, CC ligand; CCR, CC chemokine receptor; E, embryonic day; WT, wild type.
(EPS)

S1 Data A full list of genes differentially expressed between the monocyte-derived and intermediate populations. (XLS)

S2 Data Raw data used to generate each of the main figures. (XLSX)

S1 Table These are specimen data from 3 litters of E12.5 embryos, demonstrating that expression of CCL2 is only seen in monocytic cells from embryos with pre-existing expression in yolk-sac–resident monocytic cells. Yes = positive for CCL2 expression. CCL, CC ligand; E, embryonic day.
(TIF)

S2 Table List of antibodies. (DOCX)

S1 Text Additional materials and methods details. (DOCX)

## Abbreviations

ACKR: atypical chemokine receptor
BD: Becton Dickinson
CCL: CC ligand
CCR: CC chemokine receptor
Cdk4: cyclin-dependent kinase 4
cre: cre recombinase
CSF1R: Colony-stimulating factor 1 receptor
CXCL: CXC ligand
DESeq2: differential expression sequencing 2
E: embryonic day
GP49: glycoprotein 49
hCCL: human CCL
iCCR: inflammatory CCR
i.v.: intravenously
KO: knockout
LILRB4: leukocyte-immunoglobulin–like receptor B4
lrf1: liver regeneration factor 1
Lyve-1: Lymphatic vessel endothelial hyaluronan receptor 1
MFI: mean fluorescent intensity
MSD: mesoscale discovery
NK: natural killer
n.s.: not significant
Pf4: platelet factor 4
Rsad2: Radical S-Adenosyl-Methionine-Domain–Containing 2
Sca1: stem cell antigen 1
smad3: SMAD family member 3
Tgfb2: transforming growth factor beta 2
WT: wild type
YS: yolk sac
