# Supplementary figures and images for: Placental chemokine compartmentalisation: A novel mammalian molecular control mechanism
Source: PLoS Biol. 2019 May 29;17(5):e3000287. doi: 10.1371/journal.pbio.3000287 (PMC6557524; doi:10.1371/journal.pbio.3000287)

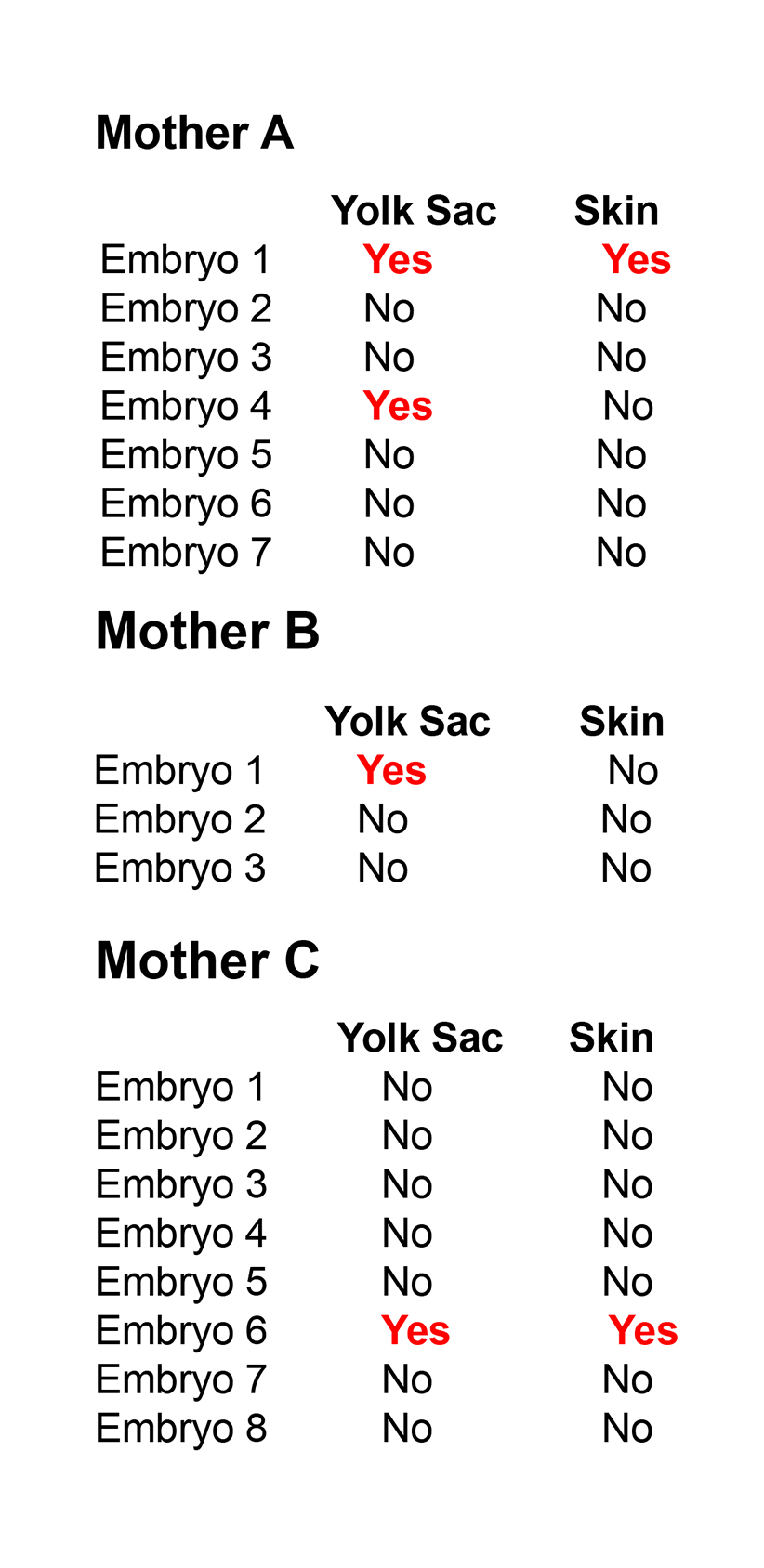

Supplement: S1 Table — Yes = positive for CCL2 expression. CCL, CC ligand; E, embryonic day. (TIF) [file pbio.3000287.s008.tif]
