# Supplementary material for: Placental chemokine compartmentalisation: A novel mammalian molecular control mechanism
Source: PLoS Biol. 2019 May 29;17(5):e3000287. doi: 10.1371/journal.pbio.3000287 (PMC6557524; doi:10.1371/journal.pbio.3000287)
Supplement: S2 Table — (DOCX) [file pbio.3000287.s009.docx]

| Antibodies | Clones | Suppliers | Usage |
| --- | --- | --- | --- |
| Anti-mouse F480-eFluor450 | BM8 | eBioscience | FACS |
| Anti-mouse CD11b-FITC | M1/70 | Biolegend | FACS |
| Anti-mouse CD11b-PE | M1/70 | eBioscience | FACS |
| Anti-mouse CD11b-  Alexa647 | M1/70 | Biolegend | FACS |
| Anti-mouse CD11b-V500 | M1/70 | BDBioscience | FACS |
| Anti-mouse CD11b-BV605 | M1/70 | Biolegend | FACS |
| Anti-mouse GP49-receptor-Alexa647 | H1.1 | Biolegend | FACS |
| Anti-mouse CD45-  AlexaAF700 | 30F-11 | Biolegend | FACS |
| Ant-mouse CD45-  AlexaAF488 | 30F-11 | Biolegend | FACS |
| Anti-mouse Ter119-  FITC | Ter-119 | Biolegend | FACS |
| Anti-mouse CD31-  Biotin | Meca13.3 | Biolegend | FACS |
| Goat anti-mouse Lyve1 | polyclonal | Biolegend | FACS/whole mount labelling |
| Chicken anti-goat IgG (H+L)-Alexa488 | polyclonal | Molecular Probe | FACS |
| Strepavidin-PerCP | N.A | Biolegend | FACS |
| Anti-mouse CD117 (ckit)-Alex647 | 2B8 | Biolegend | FACS |
| Rabbit anti-mCherry  Antibody | polyclonal | Abcam | Wholemount labelling |
| Rabbit anti-cow cytokeratin | polyclonal | DAKO | Immunohistochemistry |
| Anti-mouse CD16/32 (FcBlock) | 2.4G2 | BDBioscience | FACS |
| Chicken anti-rabbit IgG (H+L)-Alexa647 | polyclonal | Molecular Probe | Wholemount labelling |
