# Supplementary material for: Placental chemokine compartmentalisation: A novel mammalian molecular control mechanism
Source: PLoS Biol. 2019 May 29;17(5):e3000287. doi: 10.1371/journal.pbio.3000287 (PMC6557524; doi:10.1371/journal.pbio.3000287)
Supplement: S1 Text — (DOCX) [file pbio.3000287.s010.docx]

**ImageStream®X analysis of cellular morphology of mac/mono subsets in tissue digests**

E14.5 skin cell suspensions from WT embryos were analysed for morphological features at a single cell level using an ImageStreamX MarkII Imaging Flow Cytometer (Amnis, MerckMillipore) with channel-04 and channel-06 selected for Brightfield and Side scatter (SSC), both of which were disabled during compensation setup. For acquisition of E14.5 skin cell digests, laser powers were set to 120mW (Channel-01/405nm violet laser line) and 561mW (Channel-03/561nm yellow/orange laser line). E14.5 skin cells were resuspended in 200ul of PBS/2mM EDTA in 1.5ml eppendorf tubes, and then acquired and imaged at x60 magnification with a low speed. 60000 total events were acquired with the FlowSight calibration beads (Amnis, MerckMillipore) excluded from the acquisition files. Data analysis was performed on IDEAS 6.2 (Amnis, MerckMillipore). Macrophage and monocyte subsets were gated on CD11b and F4/80 from which gates of single cells were drawn based on aspect ratio and area of brightfield signals (channel-04). Single cell gates were used to generate FACS plots with contrasts_Side scatter (channel-06) vs area of brightfield (channel-04) signals for showcasing a series of morphological features of gated cells at a single cell level. Images with intact shapes were selected to generate “tagged” populations for populations of E14.5 skin (CD11bhiF4/80neg**/**monocyte, CD11bhiF4/80lo/intermediate, CD11bloF4/80hi/YS).

**RNAscope in situ hybridization detection of ACKR2**

Placentas were fixed in 4% RNAse-free PFA/PBS for 48hrs at 4ºC, followed by x3 wash in PBS for 5 min each and then stored at 4ºC until use. Fixed placentas were paraffin-embedded and used for RNAscope labelling of ACKR2-mRNA within two months post-fixation using RNAscope 2.5 High Definition (HD)—Red Assay Kit (Advanced Cell Diagnostics) and RNAscope target probes for mouse ACKR2-mRNA variant 2. RNAscope labelling on deparaffinized sections was performed according to manufacturer’s instructions, apart from the following adaptations: (1) rehydrated deparaffinized sections on glass slides were submerged into RNAscope Target Retrieval Reagents heated to, and maintained at, 95 ºC to 98 ºC (ACD) and then left for 18-19 min in the buffer followed by rinsing in distilled water immediately; (2) drops of 1:80 ratio of Fast RED-B to Fast RED-A (provided with the Red Assay Kit) were applied to cover each section for colour development. For dual-labelling of cytokeratin and ACKR2-mRNA steps using FAST RED-B and FAST RED-A were skipped. Instead, ACKR2-mRNA-labelled placenta sections were blocked in Ready-To-Use 2.5% horse serum that comes with ImmPRESS HRP Anti-Rabbit IgG (Peroxidase) Polymer Detection Kit (Vector Laboratories) for 30min at RT. Drops of rabbit anti-cow cytokeratin polyclonal antibody (Dako) or rabbit IgG as an isotype control (Vector Laboratories) diluted 1:600 in PBS/1.25% horse serum were applied to the sections and left for 1 hr at RT. Sections were washed x3 times in 0.05% PBST for 5 min each, followed by addition of Vector ImmPRESS HRP Anti-Rabbit IgG (Peroxidase) complex onto each section for 35 min at RT and, subsequent washing x3 times for 5 min each. ImmPACT SG Peroxidase (HRP) substrate complex (Vector Laboratories) was applied to each section and incubated at RT for colour development. Stained sections were washed in MilliQ water twice and then dried completely at 37ºC before mounting in EcoMount (Biocare Medical). Brightfield images were acquired on a Zeiss AxioImager M2 on a x10 lens (EC Plan-Neofluar 10x/0.30 Ph1, Zeiss).

**RNA Sequencing and bioinformatics.**

Procedures for digesting and handling E14.5 embryonic skin were the same as those for FACS analysis described above, apart from the fact that all staining for CD11b-PE (eBioscience), F480-eFluor405 (eBioscience) and CD45-AlexaFluor488 (Biolegend) was done on ice in PBS/2mM EDTA without 0.1% BSA and NaN**_3_**. Cells were labelled with Draq7 (Biostatus) to exclude dead cells.

Stained cells from each individual E14.5 embryonic skin cell digest were subjected to index sorting (single cell mode) procedure on BDAriaIII Cell Sorter (BD Bioscience) with 80um nozzle size. 100 single cells from each embryonic skin digest were sorted into 96-well PCR plates (StarLab, UK) with each well containing 4ul of lysis buffer that comes with the REPLI-g Single Cell RNA Library Kit (Qiagen). Sorted cells (final volume no more than approximately 12ul) from each embryonic skin digest were immediately vortexed quickly and spun down for 30s-45s and then subjected to digestion of genomic DNA, cDNA synthesis and amplification by multiple displacement annealing (MDA) reaction using the reagents provided with the REPLI-g Single Cell RNA Library Kit (Qiagen) in accordance with the manufacturer’s instructions. Size/length and amount of amplified cDNA products were examined and quantified by Agilent TapeStation gDNA TapsScreen test (product detection range: 200bp to 60000bp) and Qubit dsDNA HS (High Sensitivity) Assay Kit. Amplified cDNA were then purified by Agencourt AMPure XP beads (Beckman Coulter), according to the manufacturer’s instructions, and resuspended in nuclease-free water (Qiagen). Purified and amplified cDNA were kept at -20ºC until being processed for generating cDNA libraries using NEBNext Ultra DNA library prep kit. 100ng of purified and amplified cDNA were shipped to GENEWIZ (South Plainfield, NJ 07080, USA) for mechanical fragmentation and generation of cDNA libraries using NEBNext Ultra DNA library prep kit (New England Biolab/NEB). Insert size of each of the cDNA library for each sorted cell sample was approximately 400bp as quantified by Agilent 2100 Bioanalyzer using high sensitivity DNA assay. Sequencing was performed on Illumina HiSeq2500 in a 2x100bp paired-end (PE) configuration in High Output mode using V4 chemistry with a sequencing depth of ~37 million paired reads per sample (giving total ~75 million reads for each sample) by sequencing across 2 lanes.

**Downstream bioinformatics analysis of bulk RNA-sequencing (RNA-seq) data**

All bulk RNA-seq data were processed and analyzed on Linux desktop (Ubuntu 14.04.2 L). Quality controls (QCs) for the raw RNA-seq data in fastq formats were assessed using FASTQC (<https://www.bioinformatics.babraham.ac.uk/projects/fastqc/>) for standard high throughput sequence QC parameters, such as GC contents, kmer contents, N contents (non A, T, C, G bases), quality scores per base/per sequence basis, sequence duplication levels. Sequences of adaptors (New England Biolab/NEB) for read_1 (forward read of PE) and read_2 (reverse read of PE) used for DNA ligations during preparation of cDNA libraries were removed from the raw data PE fastq files using Python-based Cutadapt (<http://cutadapt.readthedocs.io/en/v1.9/installation.html?highlight=python>) with a minimum of 10bp sequence length (option –m/--minimum length) remained in the trimmed fastq files. Poor quality reads with a quality score below 25 (Sanger format) were removed from the Cutadapt-trimmed PE fastq files by Sickle command lines (<https://github.com/najoshi/sickle>). Trimmed PE fastq files were then checked for the proportion (%) of ribosomal RNA (eukaryotes: 18s, 28s, 5s, 5.8s) that were subsequently filtered out using Sortmerna command lines (<http://bioinfo.lifl.fr/RNA/sortmerna/>) based on the built-in databases of ribosomal RNA sequences provided by the Sortmerna programme. All the trimmed PE fastq files contain less than 4% of eukaryotic ribosomal RNA. Trimmed PE fastq files were then aligned to the whole UCSC mouse reference genome (mm10) using STAR Aligner[1] command lines with GTF files with Ensembl gene_IDs downloaded from UCSC Table Browser used as a reference for positions of each annotated gene in the mm10. The following STAR command lines were used for mapping of the trimmed fastq files to the mm10**:** STAR --genomeDir /directory to UCSC mm10 reference genome --readFilesIn /directory to the trimmed PE fastq files --alignIntronMax 1000000 (a default setting on the STAR) --outSAMtype BAM Unsorted --quantMode TranscriptomeSAM --outFilterMismatchNoverLmax 0.02 --outFilterMultimapNmax 5 --outFilterMismatchNmax 5 --sjdbGTFfile /directory to the UCSC GTF files with Ensembl gene IDs --outFileNamePrefix STAR/output_filename --outReadsUnmapped Fastx --outSAMprimaryFlag AllBestScore Unsorted bam files from STAR mapping outputs were inputted into Samtools[2] to generate bam files with only uniquely mapped reads and sorted bam files for data visualization on the IGV Interactive Genome Viewer (The BroadInstitute). Bam files with only uniquely mapped reads were used to obtain a gene_count_table for each of the sorted cell subsets from E14.5 skin by featureCounts (Sourceforge Susbread Package, <http://bioinf.wehi.edu.au/featureCounts/>). In-built gene annotations for mm10 in SAF (Simplified Annotation Format) embedded with the featureCounts software were used to generate gene_count_tables under meta-feature (gene_ids) with the following command lines (the default exclusion of multi-overlapping reads from counting was kept) : featureCounts –C –B –p –F SAF –R –s 1 –S fr –T 4 --ignoreDup --primary –a /subread-1.5.0-p3-Linux-x86_64/annotation/mm10_RefSeq_exon.txt (directory to the in-built gene annotations for mm10 in SAF) –o /directory to save the named output gene_count_table Aligned.out.bam (input bam file). Only unique mapped reads with both ends successfully aligned were considered for summarization in the gene_count_tables (options –B and –p). Columns for Geneids (column 1) and read counts (last column) were cut from the original gene_count_table outputs using standard Linux text editing commands to be saved as separate gene_count_tables.txt that were then loaded into R (x64 3.2.1) for statistical analysis of gene counts using R_Bioconductor bioinformatics analysis tools, DESeq2 (and edgeR) with differentially expressed genes at FDR < 0.05 considered to be significant. Lists of significantly differentially expressed genes were exported and saved as csv/excel files, in which Entrez_Gene IDs were used to identify official gene symbols from BioMart downloaded from Ensembl <https://www.ensembl.org/biomart/martview/43659a25f8f1e88bb8de2ea514f59549>. Two-dimensional (2D) Principle Component Analysis (PCA) shown on the Figure 2B depicting the transcriptional relationships between foetal WTskinR2_CD11bhiF480lo/neg (monocytic cells) and (vs) WTskinR4_CD11bhiF480lo (intermediate cells) subsets; WT WTskinR2_CD11bhiF480lo/neg (monocytic cells) and (vs) ACKR2KOskinR2_CD11bhiF480lo/neg (monocytic cells) subsets were done using built-in functions, varianceStabilizingTransformation and plotPCA (with returnData=True) provided by DESeq2. Then, 2D-PCA plots as shown on the Figure 2B were drawn on the returned data using R_CranPackage, ggplot2.

In addition, raw microarray data (Agilent) on the subsets of CD11bloF480hi and CD11bhiF480lo cells sorted from embryonic skin[3] deposited on <https://www.ebi.ac.uk/arrayexpress/experiments/E-MEXP-3510/> were imported into R for statistical analysis of differential gene expression using Limma. Differentially expressed genes between subsets of CD11bhiF480lo cells and (vs) CD11bloF480hi cells with *p* value < 0.05 were compared with those between foetal skinR2_CD11bhiF480lo/neg (monocytic cells) and (vs) skinR4_CD11bhiF480lo (intermediate cells) subsets (obtained from bulk RNA-sequencing data) to work out the number of genes overlapped together on a venn diagram using R_CranPackage, VennDiagram.

Data were deposited with the European Nucleotide Archive (Accession number, PRJEB23797).

1. Dobin A, Davis CA, Schlesinger F, Drenkow J, Zaleski C, Jha S, et al. STAR: ultrafast universal RNA-seq aligner. Bioinformatics. 2013;29(1):15-21. doi: 10.1093/bioinformatics/bts635. PubMed PMID: WOS:000312654600003.

2. Li H, Handsaker B, Wysoker A, Fennell T, Ruan J, Homer N, et al. The Sequence Alignment/Map format and SAMtools. Bioinformatics. 2009;25(16):2078-9. doi: 10.1093/bioinformatics/btp352. PubMed PMID: WOS:000268808600014.

3. Schulz C, Perdiguero EG, Chorro L, Szabo-Rogers H, Cagnard N, Kierdorf K, et al. A Lineage of Myeloid Cells Independent of Myb and Hematopoietic Stem Cells. Science. 2012;336(6077):86-90. doi: 10.1126/science.1219179. PubMed PMID: WOS:000302405400057.
